# Development of gene-in-plasmid DNA reference materials certified by single-molecule counting

**DOI:** 10.1007/s00216-024-05675-1

**Published:** 2024-12-09

**Authors:** Da-Hye Lee, Hee-Bong Yoo, Kee-Suk Hong, Sang-Ryoul Park, Sangkyun Jeong, Inchul Yang

**Affiliations:** 1 Bio-Metrology Group, Korea Research Institute of Standards and Science, Daejeon, Republic of Korea; 2 Present Address: Department of Precision Measurement, University of Science and Technology, Daejeon, Republic of Korea; 3 Quantum Optics Group, Korea Research Institute of Standards and Science, Daejeon, Republic of Korea; 4 KEYOMICS Co., Ltd., Daejeon, Republic of Korea

**Keywords:** Mole, DNA CRM, Single-molecule counting, DNA quantification

## Abstract

The mole, the SI unit for measuring the amount of a substance, was redefined as a fixed number of entities. This definition enables straightforward quantification of substances by counting individual entities. Counting proves particularly effective for quantifying large and discrete biological entities such as DNA, proteins, viruses, and cells, which are challenging to quantify via traditional physical or chemical methods. In this study, we detail our approach to develop gene reference materials certified through single-molecule counting, which enables mole-traceable measurements. We quantified three plasmid DNA constructs, each carrying a specific gene of interest, via single-molecule counting. The resulting values were cross-validated via digital PCR and LC‒MS. Sequence impurities in the certified reference materials were quantified via single-molecule real-time sequencing, whereas fragment impurities were quantified via two-color digital PCR analysis. We precisely accounted for various sources of uncertainty, including measurement precision, weighing, homogeneity, and impurities, when estimating the total uncertainty of the reference materials. In conclusion, a practical format for gene-based DNA reference materials, a measurement method to achieve metrological traceability, and methods for quantifying fragments and sequence impurities were developed and implemented in this study. We anticipate that our gene-based DNA reference materials will serve as valuable higher-order standards for the calibration of other methods or reference materials for DNA quantification in a variety of bioanalytical applications.

**Supplementary Information:**

The online version contains supplementary material available at 10.1007/s00216-024-05675-1.

## Introduction

The demand for DNA reference materials is increasing in both bioindustry and basic biological research for applications such as the detection of genetically modified organisms (GMOs) in food, testing for gene doping, molecular diagnostics, forensic DNA profiling, and advanced biopharmaceutical development [1–4]. These DNA materials must have values fitting for their intended use, via analysis of their characteristics such as their sequence, concentration, variant allele frequency, and methylation ratio.

The methods for quantifying DNA are either sequence independent or sequence specific. Optical methods, such as UV spectrophotometry and fluorometry, are sequence independent [5–7]. Traditional chemical methods, such as liquid chromatography‒mass spectrometry (LC‒MS), capillary electrophoresis, and nuclear magnetic resonance (NMR), are used to quantify DNA nucleotide content, allowing the quantification of DNA in a sequence-independent manner [8–10]. Although such methods of quantifying DNA are straightforward and well-established, sequence-independent methods are limited in that they do not discriminate nucleotides, short fragments, or non-target DNA from the DNA of interest. Thus, the demand for methods for quickly quantifying specific target DNAs is growing rapidly in modern biological, medical, and industrial applications. Examples of such applications include the detection and quantification of pathogens, GMOs, and nucleic acid impurities in biological goods. Most of the optical and chemical analyses mentioned above are unsuitable for such applications.

PCR and next-generation sequencing (NGS) are well-known methods of sequence-specific DNA analysis. The combination of quantitative PCR (qPCR) and UV spectrophotometric results has been used to quantify intact genes or sequence-specific DNA [11]. Recently, reference values have been assigned via digital PCR, which detects the amplified fluorescence signal of single DNA molecules in many individual partitions, eliminating the need to generate a standard curve [12]. However, the reference values obtained via both qPCR and digital PCR depend on amplification, which is affected by the GC content of the template DNA, the sequences and quality of the primers and probes, the salt concentrations, the reaction mixture pH, the Taq polymerase enzymatic activity, and other factors [13–15]. Thus, PCR-independent DNA quantification techniques are necessary to validate PCR efficiency [16–18]. DNA reference materials with higher metrological validity will be helpful in verifying and calibrating other more conventional methods of DNA quantification.

There are several advanced technologies that can be used for directly counting individual DNA molecules. For example, one group developed a method for using a microscope to count fluorescence-labeled DNA molecules attached to a microchannel surface [19]. Another report described super-resolution microscopy-based counting of surface-fixed DNA molecules [20]. Another report showed that solid-state nanopores could be used to capture and quantify single DNA molecules [21]. We also developed and reported a method using capillary flow cytometry to count individual nucleic acid molecules. This method is PCR independent and employs single-molecule counting of fluorescence-labeled DNA or RNA flowing through a capillary [22–24].

In this work, we report our preparation and certification of gene reference materials via a single-molecule counting-based DNA measurement method with traceability. Our newly developed reference materials carry the human adenovirus type 5 (hAd5) E1 gene, a partial human PSG4 (pregnancy specific beta-1-glycoprotein 4) gene adjacent to hAd5 E1, and the SARS-CoV-2 S gene. To assess the homogeneity and short-term stability of the reference materials, we used a single-molecule counting method. We used single-molecule NGS and two-color digital PCR to analyze the reference materials for impurities, such as fragmented plasmids and sequence-mismatched DNAs. Uncertainties were evaluated, considering measurement precision, weighing, homogeneity, and impurities as major sources of uncertainty.

Thus, our certified reference materials (CRMs) can be used to calibrate PCR instruments for qPCR or digital PCR applications. Moreover, our CRMs can be used as quantitative positive controls for adenovirus type 5 or adenovirus-based therapeutics and for quantifying SARS-CoV-2 or analyzing SARS-CoV-2 vaccines. These DNA reference materials with high metrological validity will be helpful in verifying and calibrating conventional methods of DNA quantification.

## Materials and methods

### Plasmid DNA preparation

The human adenovirus type 5 E1 gene was PCR-amplified using specific primers (F: 5′-GATGACTTGTCCATCATCAATAATAT-3′ and R: 5′-TGGCAATCAGCTTGCTACTGAAAG-3′). To include human genomic DNA adjacent to the Ad5 E1 gene [25], PCR was performed with the appropriate primers (F: 5′-TCCCATGGAATCACGAAGAG-3′, R: 5′-GGAATGAGCTGCATATCAGGA-3′). We added the T7 promoter sequence, 5′ and 3′ UTR sequences, and a poly-A tail to the SARS-CoV-2 S gene via PCR using specific primers (F: 5′-taatacgactcactatagGGGAAATAAGAGAGAAAAGAAGAGTAAGAAGAAATATAAGAGCCA-CCATGTTTGTTTTTCTTGTTTTATTGCCACTAG-3′, R: 5′-GGAGGGGCTGGGGGGAGGCCCAAGGGG-CAAGAAGCATGGCCACCGAGGCTCCAGCCTATTATCATTATGTGTAATGTAATTTGACTCCTTTGAGC-3′, R2: 5′-TTTTTTTTTTTTTTTTTTTTTTTTTTTTTGCCGCCCACTCAGACTTTATTCAAAGACCACGG-GGGTACGGGTGCAGGAAGGGGAGGAGGGGCTGGGGGGA-3′). All PCRs were performed using KAPA HiFi polymerase (Roche) to minimize errors. The PCR products were inserted into the pTOP vector (Enzynomics) by TA cloning, and the selected clones were subsequently verified via Sanger sequencing. A sequence-confirmed plasmid was then mass produced in DH5α *E. coli*. The total plasmid DNA concentration was measured with a UV spectrophotometer. Then, 35–50 μg of the three plasmid DNAs were linearized via incubation with a restriction enzyme (XhoI, NEB R0146M) for 2 h at 37 °C. Finally, the enzyme was heat-inactivated for 20 min at 65 °C.

### Electrophoresis and mass spectrometry

The approximate quantities of the plasmid DNA samples were estimated using a NanoDrop™ 2000 spectrophotometer (Thermo Fisher). DNA sizes and impurities were evaluated via TapeStation™ 4150 agarose gel electrophoresis (Agilent). The genomic DNA ScreenTape™ kit was used because the plasmid DNA was over 7 kb in length. The total DNA quantities of the CRMs were measured via isotope-dilution mass spectrometry (LC‒MS) using Korean Research Institute of Standards and Science (KRISS) dNMP reference materials as calibrators and stable isotope-labeled DNA (SILD) as an internal standard [10]. In brief, 10 ng of plasmid DNA samples was gravimetrically mixed with SILD internal standards and then enzymatically hydrolyzed via Nucleoside Digestion Mix™ (NEB), and the KRISS dNMP reference materials were processed under exactly the same conditions in parallel. The hydrolyzed samples were diluted tenfold in distilled water and subjected to LC‒MS/MS analysis. The samples were analyzed on a Zorbax SB-Aq™ column (Agilent) with an isocratic mobile phase of 20% acetonitrile supplemented with 0.1% formic acid using a 1200 series LC system™ (Agilent). Nucleosides were detected in positive MS/MS mode using an API4000™ mass spectrometer (SCIEX). For LC‒MS/MS, all samples were gravimetrically prepared from three independent bottles and analyzed in triplicate.

### Droplet digital PCR

For droplet digital PCR, samples were gravimetrically diluted to 1300 copies/μL, and 2 μL of each diluted sample was volumetrically added to the PCR mixture. All the digital PCR mixtures (Table 1) included 1 µM primers, 250 nM probe, and 1X ddPCR Supermix (Bio-Rad). The reaction mixtures were subsequently loaded into an automated droplet generator (Bio-Rad). The generated droplets in a 96-well plate were placed in a thermocycler (Applied Biosystems Veriti96). The PCR conditions were 10 min at 95 °C followed by 70 cycles of 30 s at 94 °C, 1 min at 60 °C, and 10 min at 98 °C, with a ramp rate of 2 °C/s. Ten independent bottles per CRM were analyzed via dPCR, and each dPCR was performed in triplicate.

**Table 1:** Primers and probes

Oligo name	Orientation	Sequence
vector	F	AACGTCGTGACTGGGAAAAC
	R	CGGGCCTCTTCGCTATTAC
	HEX	TAATCGCCTTGCAGCACATC
E1	F	CTGTCACCTGCTGAAGACCA
	R	GGAACAGCGGGTCAGTATGT
	FAM	TCACGTAGCCAGCCACTCTC
E1-PSG	F	GAACCACCTACCCTTCACGA
	R	CAGAGTCGGGAAAAATCTGC
	FAM	ATTTAGACGTGACGGCCCC
S	F	CTTCCCTCAGTCAGCACCTC
	R	GACAAATGGCAGGAGCAGT
	FAM	GCATGTGACTTATGTCCCTGCA

### Single-molecule counting

The prepared plasmid DNAs were verified by single-molecule counting in a custom capillary flow cytometer [22]. For counting, the DNA samples were diluted to a final concentration of approximately 2000 molecules/μL in flow cytometer (FCM) running buffer (5 mM Tris–Cl, pH 9.5) through two rounds of gravimetric dilutions. Five microliters of 0.1X SYBR Gold dye (Thermo Fisher) was added to each 1 mL final sample. The samples were then mixed by vortexing for 10 s and allowed to rest for 15 min at room temperature. Dilutions and incubations were performed using borosilicate vials (Agilent 5182–0716) to minimize on-process adsorption of diluted DNA. The procedure and technical details for single-molecule counting using a capillary flow cytometer were described in previous reports [23]. In brief, SYBR Gold-stained DNA samples were used to overfill a 50 μm × 50 μm square capillary tube (Molex), with a predetermined volume of 1.44 μL. For determination of the sample volume in the capillary tube, water was used to fill a 10-m capillary tube. Next, the water in the capillary tube was removed by the overinjection of air via a syringe pump. Eluted water from the capillary tube was collected and weighed. The difference (24.2 mg) in weight represents the sample volume (24.3 μL, assuming a density of water of 0.997 at 25 °C) in a 10-m capillary tube. The capillary volume determined in this way was equivalent to the volume calculated from the manufacturer’s specifications within 1%. The distance between the inlet end and center of the detection window of the capillary tube was measured as 59.3 cm. On the basis of these values, the samples in the capillary tube and exhaustively analyzed by counting were calculated to have a volume of 1.44 μL. A pressure of 0.07 MPa and an electric field (−5 kV) were used to move the samples slowly toward the capillary outlet. The combined application of pressure and an electric field ensures that DNA molecules focus and move along the center of the capillary. Measurements were performed for 120 s, while more than 99% of the DNA molecules passed through the detection window within 60 s. DNA molecules passing through the detection window were counted with a laser-induced fluorescence detection system. Photon signals collected by the avalanche photodiode-based photon counter (Excelitas; SPCM-AQRH-16-FC) were transmitted to a computer through the USB-6001 I/O device (National Instruments). Our single-molecule counting system was implemented with new in-house signal acquisition and data processing software. The in-house software was developed on the basis of Visual Studio™ and the C# language. Configuration of operational options and instrumental controls, real-time data display, and the analysis of transmitted photon signals were performed in the software.

### Single-molecule NGS

Single-molecule NGS was performed through a commercial service (Macrogen, Korea) based on the Sequel II single-molecule real-time sequencing (SMRT™) system (PacBio). One microgram of each DNA sample was processed with a SMRTbell prep kit 3.0 (PacBio) to generate NGS libraries. Only consensus circular reads (CCSs) from SMRT sequencing were collected for further analysis. The collected CCSs were aligned with the predefined sequences of the three plasmid DNA constructs via a BLAST algorithm for the identification of fragments, mutations, and sequencing errors. Sequences that were not aligned to the authentic plasmid construct sequences were aligned to other databases, including the human genome, bacterial genomes, and viral genomes, to identify their origins. Sequences aligned to these off-target databases were considered sequence impurities in the CRMs.

## Results

### Strategy for developing gene reference materials

In this study, we aimed to demonstrate the effectiveness of a strategy for developing certified gene-based DNA reference materials that are traceable to the definition of “mole.” The gene-based DNA reference materials used in this study were produced via three distinct processes. First, raw DNA materials with predefined sequences and lengths were prepared. Second, the prepared materials were quantified via single-molecule counting technology. Finally, the certified reference materials were further characterized with respect to impurities, stability, and homogeneity to estimate the relevant measurement uncertainties.

**Fig. 1:**
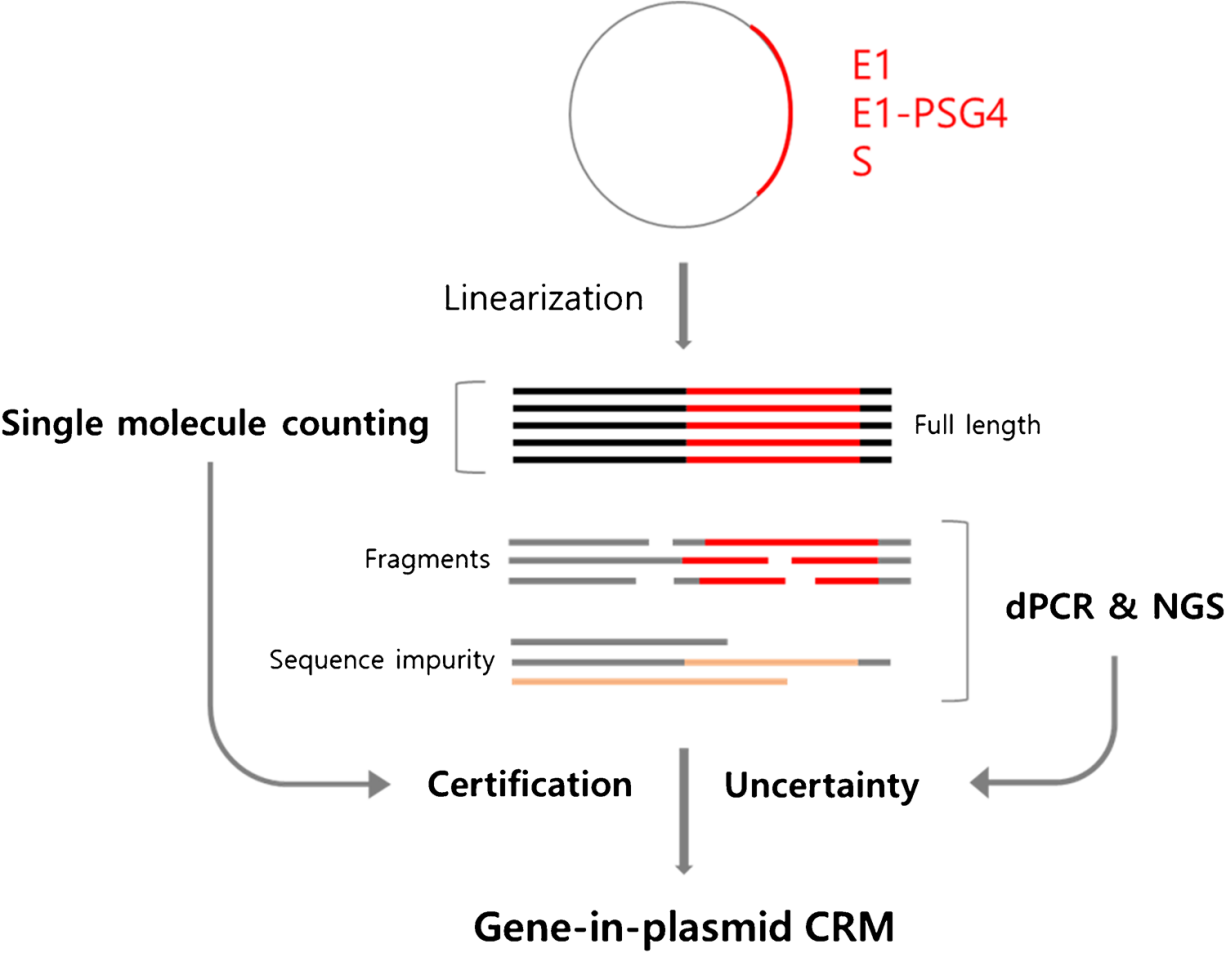
Schematic for the certification of plasmid CRMs by single-molecule counting. Only full-length DNA with the correct sequence was considered an authentic measurand. Fragments and DNA with different sequences were considered impurities

Figure 1 illustrates the overall concept and the workflow used to produce the gene-based DNA reference materials used in this study. Our single-molecule counting technology was validated for accurate quantification of 3–50 kb DNA [22–24]. The target DNA needs to be of a regular length to avoid misinterpretation of the data, since the technique itself does not accurately discriminate DNAs of different lengths. The concentration of DNA needs to be above 10^4^ molecules per μL, and DNA at higher concentrations is gravimetrically diluted before being counted. Notably, only full-length DNA molecules with the correct sequence were considered authentic measurands. The authentic measurands and full-length DNA molecules were quantified via single-molecule counting. On the other hand, DNA with different sequences or DNAs of different lengths were considered impurities. Single-molecule next-generation sequencing (NGS) and two-color digital PCR analysis were performed to specifically quantify sequence impurities and fragment impurities, respectively. The proportions of impurities were considered in the estimation of the uncertainty of the CRMs.

### Preparation of raw materials and preverification of pilot batches

**Fig. 2:**
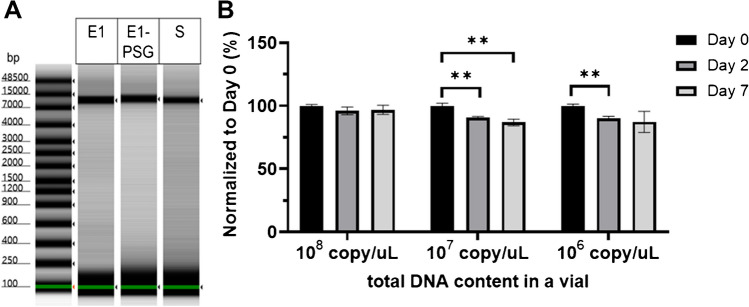
Preliminary tests of the raw materials and certification conditions. **A** Electrophoresis of linearized plasmid DNA. **B** Preliminary tests of the short-term stability of candidate plasmid DNA RMs at different concentrations. **p value < 0.05

PCR-amplified genes of interest were cloned and inserted into plasmids and mass produced as raw materials for CRMs. A pilot batch of raw materials was prepared for a few preliminary tests. The pilot batch of plasmid DNA was confirmed by Sanger sequencing (Supplemental Fig. 1), electrophoresis, and UV spectrophotometry. The plasmid DNAs were linearized by restriction enzyme digestion, since circular or supercoiled plasmid DNA structures reduce PCR efficiency [26] and result in variable fluorescence intensity during single-molecule counting. Linearized plasmid DNA was analyzed by electrophoresis at TapeStation to assess the completeness of linearization, fragmentation, and the inclusion of impurities (Fig. 2A). We did not observe any significant sign of fragmentation or the inclusion of impurities in our preliminary analysis of the candidate plasmid DNA. Next, the concentrations of the DNA CRMs were determined. CRMs with very high concentrations will accordingly require large amounts of raw materials, leading to higher costs. In contrast, RMs with lower concentrations could be accompanied by homogeneity and stability issues due to the possibility of DNA adsorption to the plastic vial and increased degradation [27, 28]. We tested the short-term stability of three different concentrations of candidate plasmid DNA CRM batches ranging from 10^6^–10^8^ copies/μL based on UV spectrophotometry. For the 8 kb plasmid DNA, 10^8^ copies/μL translated to approximately 1 ng/μL. As shown in Fig. 2B, samples at all three concentrations were successfully quantified via single-molecule counting. The quantified values were consistent with the estimates obtained by UV spectrophotometry on day 0. However, a reduction in values was observed on days 2 and 7 for the samples with 10^6^ copies/μL and 10^7^ copies/μL, respectively, during storage at 25 °C, whereas the values for the samples with 10^8^ copies/μL remained stable. These decreases in the samples with lower DNA concentrations are likely due to either increased DNA degradation or increased DNA adsorption. On the basis of these data, we decided to prepare our gene-based DNA reference materials at a concentration of 10^8^ copies/μL.

### Measurement and data analysis

**Fig. 3:**
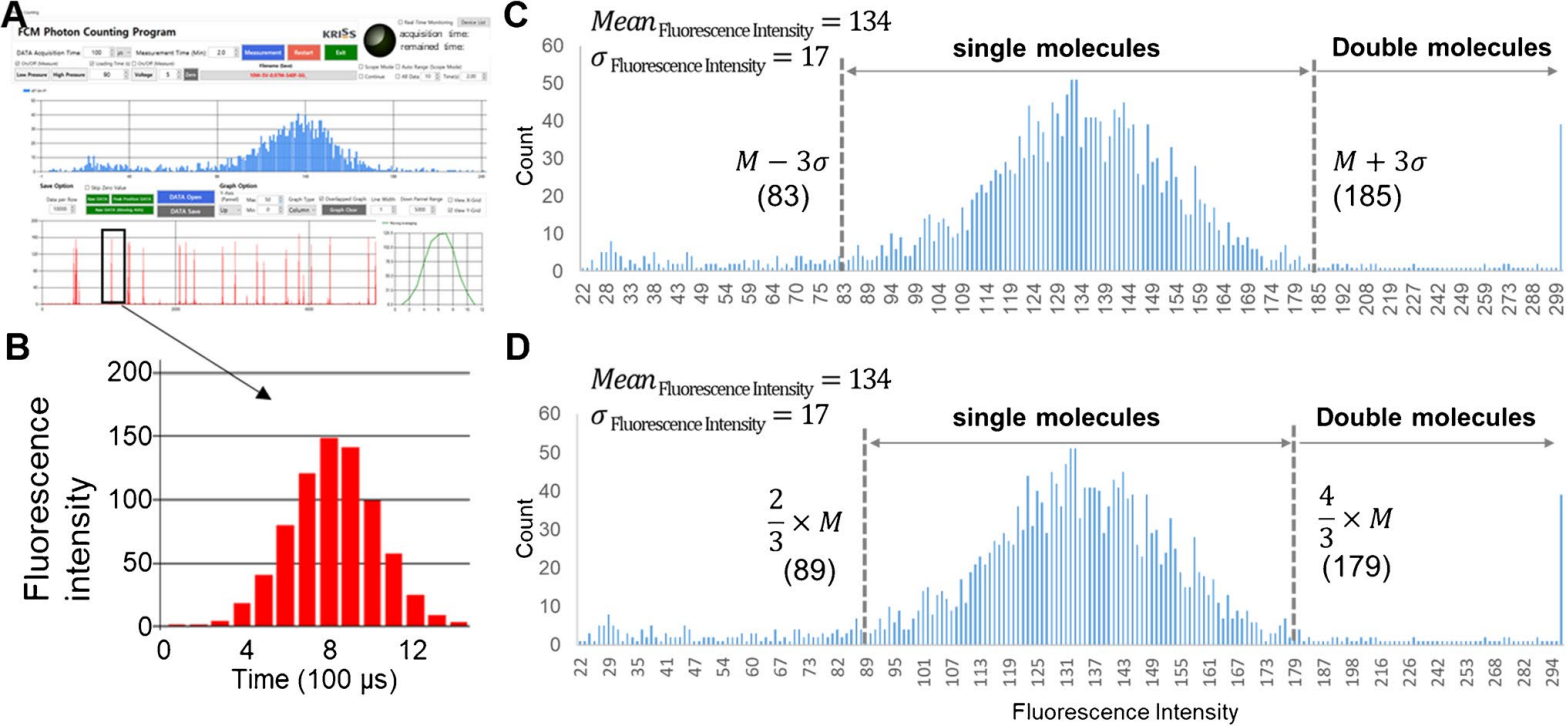
Representative data acquisition and processing for the S gene CRM. **A** Raw data processing via the in-house photon counting system. Photon signals were collected at 100 μs intervals. **B** Magnified raw photon counting results for a single DNA molecule. **C** $\text{Mean}\pm 3\upsigma$ threshold setting. **D** An alternative threshold setting strategy. M; mean, σ; standard deviation

We prepared and certified three different plasmid DNA reference materials carrying the adenoviral E1 gene, the adenoviral E1-human PSG4 fusion gene, and the SARS-CoV-2 S gene. Certification was performed by counting single molecules as they passed through a capillary flow cytometer. Figure 3A shows a screenshot of our in-house single-molecule counting system, where raw photon counts are collected, displayed, and analyzed. Photon signals collected at 100 µs intervals are transmitted and displayed as red peaks. A magnified peak from a single 8 kb plasmid DNA molecule is shown in Fig. 3B. The typical peaks representing single DNA molecules have heights of approximately 140 photons and durations of approximately 0.8 ms. Overlap of DNA molecules could result in either higher peaks (Supplemental Fig. 2a) or longer durations (Supplemental Fig. 2b). We used specific criteria to identify peaks that needed to be counted as double molecules in our photon counting system. For example, peaks higher than 186 photons or wider than 1.2 ms were considered double-molecule peaks for the S gene reference materials. These double-molecule peaks accounted for 3–6% of the total DNA count.

We considered only full-length plasmid DNA constructs as valid measurands for certification, discarding DNAs fragmented to sizes below a certain threshold. When using this measurand definition and certification principle, we needed to detect and count all the DNA molecules that flow through the capillary. Then, we needed to set the discarding threshold as low as possible to minimize the loss of the true DNA signal. This strategy, however, increased the chance of overestimation by including DNA fragments that do not fit our definition of a valid measurand. As a compromise, we set the threshold for the acquisition of a valid signal at $\text{M}\pm 3\upsigma$ (Fig. 3C, M, mean of the distribution; $\upsigma ,\text{ standard deviation}$). The resulting lower thresholds corresponded to 5.3 kb, 5.6 kb, and 4.6 kb for the E1, E1-PSG4, and S plasmids, respectively. Peaks falling beyond the upper threshold were considered DNA molecule overlaps and were counted as 2 per peak. An alternative strategy for setting the threshold could be based on two assumptions. First, the distribution must be symmetric. Second, the lowest signal intensity for a double-molecule peak (i.e., an overlap of two molecules) would be numerically double the lowest signal intensity of a single-molecule peak. On the basis of these assumptions, if we set ($\text{M}-\updelta$) as the lower threshold for single-molecule peaks and ($\text{M}+\updelta$) as the upper threshold, $2\times (\text{M}-\updelta )$ would be the lower threshold for double-molecule peaks. Since the higher threshold for a single-molecule peak needs to be the same as the lower threshold for a double-molecule peak, ($\text{M}+\updelta$) would be equal to $2\times (\text{M}-\updelta )$. This would mean that $\updelta$ would be calculated as $\frac{1}{3}\times \text{M}$. Then, the peaks from $\frac{2}{3}\times \text{M}$ to $\frac{4}{3}\times \text{M}$ are counted as single molecules, and the peaks higher than $\frac{4}{3}\times M$ are counted as double molecules (Fig. 3D). We found that the certified values from these two different threshold-setting strategies were equivalent within a 1.2% margin of error. Since the two threshold-setting strategies did not lead to significant differences, we based our current certified values on the first strategy using the “M ± 3σ” thresholds. We expect to employ the latter strategy in future CRM certification after further validation.

### Certification and evaluation of homogeneity and stability

**Fig. 4:**
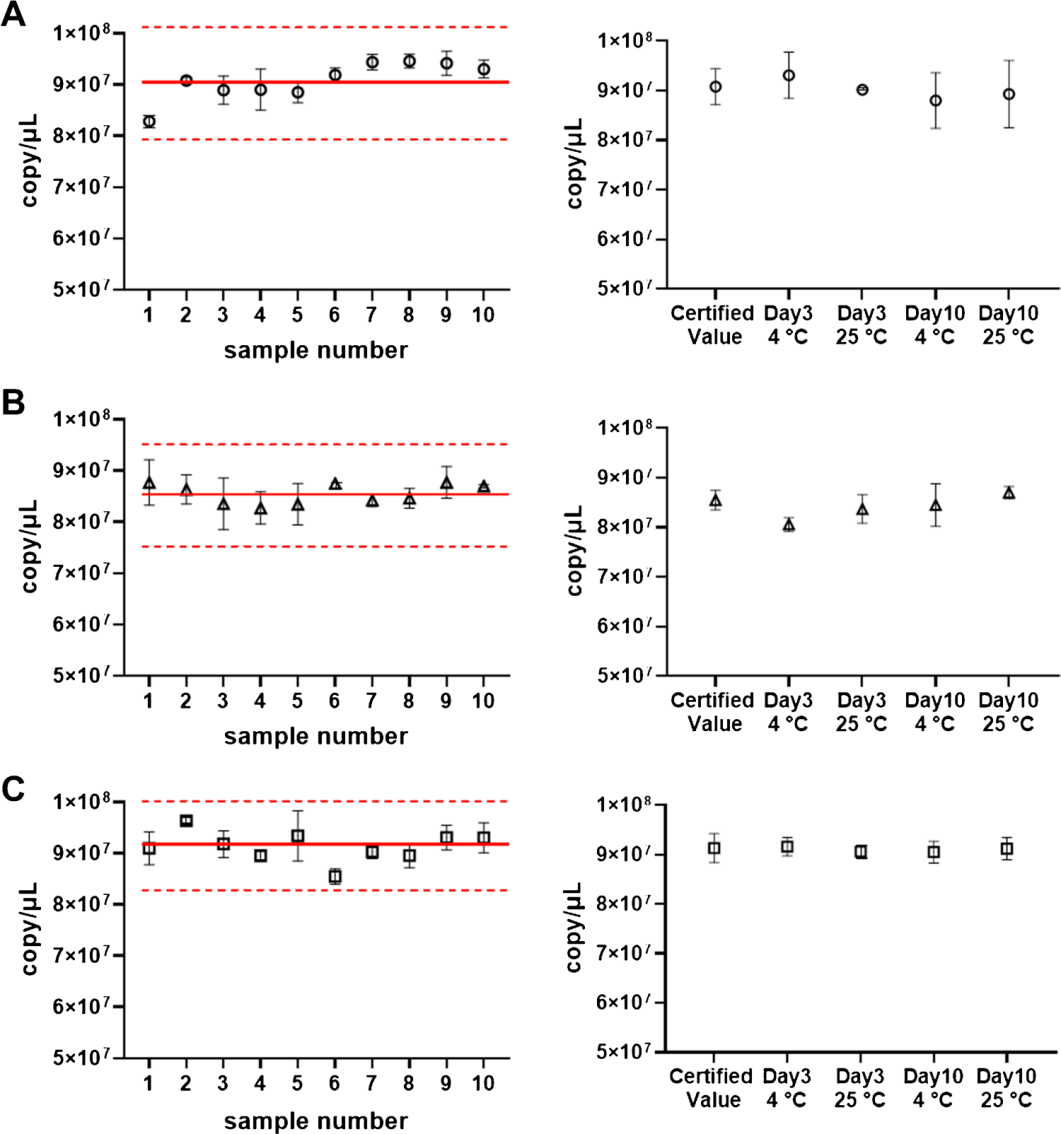
Homogeneity and short-term stability of the CRMs. The results for CRMs of the E1 (**A**), E1-PSG4 (**B**), and S (**C**) plasmids. The left panels indicate homogeneity, whereas the right panels indicate short-term stability. The solid and dotted lines represent the certified values and expanded uncertainties, respectively

We evaluated CRM homogeneity with measurements from ten independent bottles and short-term stability from samples stored for up to 10 days at 4 °C and 25 °C (three bottles per storage condition). The results are shown in Fig. 4. ANOVA tests were performed to evaluate the between-bottle homogeneity of the CRMs. The results of ANOVA indicated that the E1 and S CRMs may not be homogeneous in statistics. Despite the indications by the ANOVA tests, we concluded that our CRMs are acceptable based on ISO guidelines for checking the homogeneity of reference materials [29]. The relative standard deviations (CV) of the values from 10 independent bottles were 4.0% for E1, 2.3% for E1-PSG4, and 3.2% for S. These CVs were around one-thirds of uncertainties of the CRMs and considered acceptable based on the ISO guideline. No statistically significant change in DNA quantity was observed during the short-term storage of the CRMs (Fig. 4, right panels). On the basis of these results, we concluded that all three CRMs are homogeneous within their range of uncertainty and sufficiently stable for 10 days under the tested temperature conditions.

### Impurity analysis

**Fig. 5:**
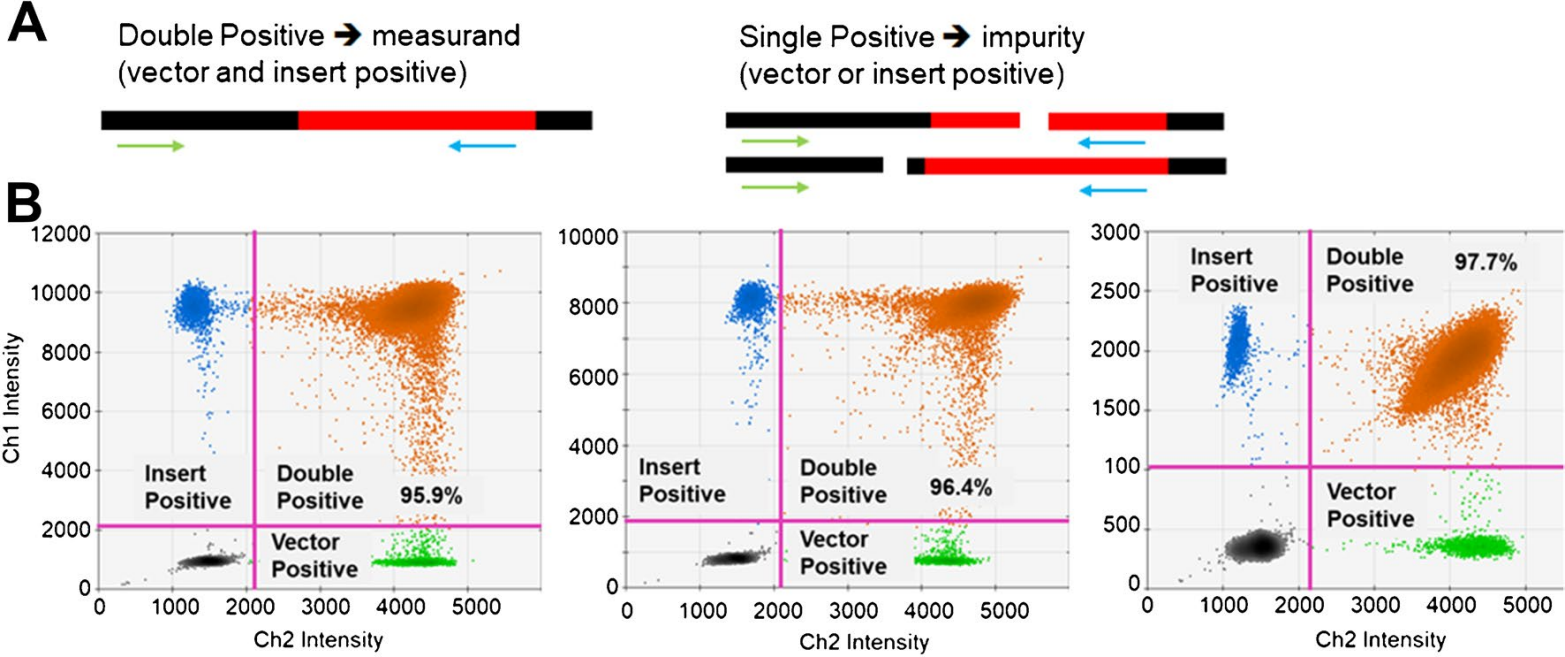
Quantification of DNA fragments in the CRMs. **A**. The concept for measurement of fragment impurities in the CRMs by 2-color digital PCR assays.  **B** A representative 2D plot and threshold setting for QuantaSoft analysis.

Two-color digital PCR analyses were performed to quantify not only full-length DNA molecules but also the fraction of fragmented DNA. Two-color digital PCR has been applied for linkage analysis to determine whether two genes are close to one another [30]. Under typical droplet-based digital PCR conditions, two independent DNA fragments are unlikely to be compartmentalized in the same droplet. If two genes are amplified with fluorescence of different colors, fluorescence of two colors will be observed in a single droplet only when the two genes are physically contiguous. We designed two amplicons with different colored probes near the 5′ (vector region) and 3′ (insert region) ends of the DNA reference materials (Fig. 5A). Molecules that remain unbroken will produce double-positive droplets. In contrast, breaks in the molecules separate the two PCR targets in different droplets, leading to single-positive droplets. Thus, this technique allows the simultaneous quantification of full-length DNA and fragmented DNA with double-positive and single-positive signals, respectively. The results from our two-color digital PCR analyses for the three CRMs are shown in Fig. 5B. We used the copy number concentrations obtained from double-positive droplets as informative values for the CRMs. We found that the concentrations of the three reference materials measured by digital PCR were equivalent to the certified values measured by single-molecule counting. The average proportions of single-positive (vector-positive and insert-positive) droplets were 4.8% (E1), 4.6% (E1-PSG), and 2.4% (S). These proportions indicating breakages between the two PCR targets were interpreted as fragment impurities in the reference materials. However, it should be noted that fragments quantified by two-color digital PCR are a minimal estimate of fragments since the method involves the detection of only amplifiable fragments. Very short fragments that do not include the full amplification targets of digital PCR cannot be detected via this method. It is also possible that DNA molecules with sequence variations or modifications that hinder PCR amplification cannot be detected via this approach.

**Fig. 6:**
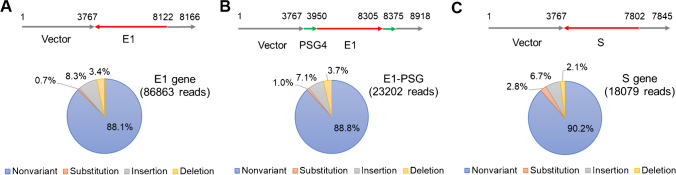
Schematic diagrams of the DNA structures and pie charts depicting the proportions of sequence variants in the CRMs. The image at the top of each panel represents DNA structure.  Gene inserts are indicated by numbers. Arrows indicate sequence directions. Grey: vector. Red and green: insert. Compositions of sequence variants determined by single-molecule sequencing are shown as pie charts.

We next performed single-molecule real-time (SMRT™) sequencing via the PacBio™ sequencing platform to obtain the consensus sequences and structures of the DNA reference materials, as well as information on sequence impurities, such as fragments, variants of the desired reference sequences, and bioprocess-related sequence impurities (e.g., host cell DNA) [31]. SMRT™ sequencing is better than shotgun sequencing for identifying fragment impurities and the distributions of small variants because it produces self-confirmed long reads from single molecules of DNA. Normal shotgun NGS does not discriminate fragments from full-length DNA sequences, nor does it retain any topology information about sequence variations in a single molecule. Figure 6 shows the structures of the reference materials and the proportions of sequence variants, as determined by NGS. Non-variant sequences comprised 88–90% of the reference material, with the remainder comprising sequence variants such as substitutions, insertions, and deletions. A closer look at the sequence variants, however, revealed that most deletions and insertions had the same bases at the end of homonucleotide stretches. Many reports have indicated that homonucleotide stretches often lead to Sanger and NGS sequencing errors that appear as deletions or insertions of the same bases [32, 33]. Therefore, a significant proportion of the small variants in our CRM may have arisen from simple sequencing errors rather than from true sequence variants. This would mean that the proportions of non-variant sequences in the CRMs are minimum values for the authentic plasmid DNAs.

In addition, we identified sequence impurities that exhibited no homology to the reference materials. More than 99% of these sequence impurities were aligned to either the *E. coli K12* genome or the *E. coli DH5α* genomes (Table 2). The *DH5α*-aligned sequences were the major sequence impurities originating from host bacteria because *DH5α* was used to produce the plasmid DNA. On the other hand, we did not consider the *K12*-aligned sequences to be sequence impurities in the reference materials but rather simple analytical artifacts. The *K12* strain is widely used for the production of the enzymes used in the manipulation of nucleic acids. The use of such enzymes during NGS library preparation could result in the observation of *K12* genomic DNA in NGS data, which is not a true representation of impurities in the actual reference materials. Although sequence impurities from *E. coli DH5α* were estimated to account for 1.8% (E1), 1.8% (E1-PSG), and 1.3% (S) of total DNA contents in the CRMs, only 0.8%, 0.8%, and 0.6% of impurities that were longer than the thresholds for counting were accounted for in the calculation of uncertainty.

**Table 2:** Uncertainty from sequence impurity

CRM	Total reads	DH5 reads	K12 reads	DH5 & K12 reads	Total DH5 reads	DH5 impurity	Impurity over threshold
E1	96,137	440	1829	6546	1709	1.8%	0.8%
E1-PSG	26,298	124	633	2142	475	1.8%	0.8%
S	19,774	67	386	1314	261	1.3%	0.6%

## Discussion

### Traceability

The mole has been redefined as a fixed number of entities. This means that simple counting of individual entities can lead directly to mole-traceable quantities of substances. Counting is particularly beneficial for the quantification of biological entities that are both discrete and sufficiently large. Our strategy to develop mole-traceable gene-based DNA reference materials involves the preparation of a DNA construct harboring a gene of interest and counting of the individual DNA constructs. One drawback of counting DNA molecules is the difficulty with which target DNAs of interest in the sample can be distinguished from irrelevant entities. Thus, to achieve mole-traceable DNA measurements, it is important to prepare a homogenous DNA sample with minimal nucleic acid impurities. We defined only the designed construct with the correct sequence, structure, and length as a valid measurand in this study. Then, RNA, DNA with irrelevant sequences, and DNA fragments, even those with correct sequences, could all be considered impurities that could hamper the realization of molar traceability. In this context, we employed two new methods to quantify major impurities in the CRMs. Two-color digital PCR was performed for the measurement of fragmented DNA, which is not considered a valid measurand, and single-molecule NGS was used for the identification and quantification of sequence impurities. The percentage of fragment impurities in the CRMs was estimated to be approximately 2.4–4.8%, whereas the percentage of sequence impurities was 0.6–0.8%. The impurities were accounted for in the uncertainties of the CRMs, which provides margins for traceability. The use of plasmid DNA constructs is highly beneficial in the preparation and certification of gene-based DNA reference materials. DNA in plasmid constructs is easy to handle and can be efficiently mass produced and purified. Any genes of interest can be inserted into the plasmid constructs and developed as gene-based DNA CRMs through a valid certification method. Our strategy to certify mole-traceable plasmid DNA reference materials via single-molecule counting automatically leads to the mole-traceable certification of the genes in the plasmid.

### Uncertainty

Mole-traceable measurements of DNA are critical for assessing the purity of a sample. In our certification strategy, all entities that are fluorescently activated by binding with SYBR Gold are considered impurities. RNA and DNA with different sequences are major impurities in our gene-based DNA reference materials. DNAs of different sizes, even those with authentic sequences, are also considered impurities. It would theoretically be best to eliminate all such impurities in the preparation of raw materials for the CRMs, but in practice, this is nearly impossible. It is also possible to reintroduce fragment impurities even with careful handling of the raw materials. We classified those impurities that were not completely eliminated as major sources of uncertainty for the developed gene-based DNA reference materials.

CRM uncertainty was assessed considering the effects of various parameters on two major procedures, preparation and certification (Table 3). Sources of uncertainty that arise during CRM preparation include within-bottle homogeneity, between-bottle homogeneity, stability, fragment impurities, and sequence impurities. For our CRMs, the uncertainty arising from within-bottle homogeneity issues was negligible because it was not discriminable from the standard uncertainty reflecting the repeatability of the measurements. The between-bottle homogeneity was estimated from the standard deviation of values derived from the certification of ten independent CRM bottles. The uncertainty arising from short-term stability issues was also negligible because we were unable to observe any statistical signature of instability during short-term storage. One of the largest sources of uncertainty for our current gene-based DNA CRMs, fragment impurities, was evaluated via two-color digital PCR assays. While the measured fragment impurities were 4.8% (E1), 4.6% (E1-PSG), and 2.4% (S), only proportions that are longer than the thresholds for counting (3.2%, 3.4%, and 1.5%, respectively) were accounted for fragment uncertainty (Table 3). The uncertainty arising from sequence impurities was 0.6–0.8%, with most of the sequence impurities comprising host *E. coli* genomic DNA fragments. This contamination with *E. coli* genomic DNA was inevitable because the raw plasmid DNA was prepared in *E. coli* cultures and further processed using restriction enzymes produced in *E. coli*.

**Table 3:** Breakdown of uncertainty of the CRM

Source	Type	Assessment/interpretation	Estimated values (%)
RM preparation			
Homogeneity (within bottle)	Random	Three samples from a bottle Indistinguishable from repeatability	1.2 (E1), 1.8 (E1-PSG), 1.4 (S)
Homogeneity (between bottle)	Random	Standard uncertainty from measurement of 10 independent bottles	1.3 (E1), 0.7 (E1-PSG), 1.0 (S)
Stability	Systematic	Short-term stability: not significant Long-term stability: planned to be tested	Not determined
Fragment impurity	Systematic	Proportion of fragment impurities measured by two-color dPCR over thresholds for counting (4.8–5.6 kb)	3.2 (E1), 3.4 (E1-PSG), 1.5 (S)
Sequence impurity	Systematic	Proportion of sequence impurities assessed by “Single-molecule NGS” over thresholds for counting (4.8–5.6 kb)	0.8 (E1), 0.8 (E1-PSG), 0.6 (S)
RM certification			
Weighing	Systematic	Estimated based on the balance manufacturer’s specification	3.0 for all
In-process adsorption and fragmentation	Systematic	Possibility of molecular adsorption or fragmentation during sample preparation and counting	Not determined
Repeatability	Random	Three independent replicates of processing and measurement for a single sample	1.7 for all
Assignment of sample volumes	Systematic	Variation in determining sample volumes introduced into the capillary (weighing, electron microscopy and vendor’s specifications)	0.7 for all
Signal and data processing	Systematic	Probability that intact DNA molecules will not be detected with the 3σ–threshold setting	0.6 for all
Detection failure	Systematic	Needs for a better reference method or reference materials Equivalence with dPCR results: not significant	Not determined

Expanded uncertainty (95%, *k* = 2): 11.2% (E1), 11.1% (E1-PSG), 8.3% (S)

We also considered several other potential sources of uncertainty during certification, including weighing, counting repeatability, in-process fragmentation, in-process adsorption, sample volume determination, data processing, and failure of DNA detection. Among them, weighing was the greatest source of uncertainty because we weighed very small amounts of DNA (4 mg) in the preparation of diluted samples for counting. To reduce the uncertainty arising from weighing, we recommend preparing larger amounts of the samples by gravimetry and using only a tiny fraction of the prepared samples for measurements.

The uncertainty arising from sample volume determination was estimated to be as low as 0.6%. Routine biological measurements such as digital PCR and flow cytometry are generally associated with relatively large uncertainties arising from sample volume determination. Our single-molecule counting system measures predefined sample volumes in a capillary, which minimizes sample volume uncertainty [34]. We did not estimate uncertainties arising from in-process fragmentation, adsorption, or detection failure in this study because well-established orthogonal analyses or certified reference materials would be required for the evaluation of those fundamental parameters affecting the biases and uncertainties of measurements.

Overall, we estimate the expanded uncertainty of our CRMs to range from 8.3 to 11.2%, which we regard as satisfactory because these are the first mole-traceable gene-based DNA CRMs. We acknowledge the need to improve our DNA purification process and fragment analyses if we want to produce higher-quality DNA CRMs because the uncertainty arising from our DNA preparation process was greater than that arising from our certification process. Because we did not assess the uncertainty arising from detection failure, in-process fragmentation, and in-process adsorption, further studies are needed to determine how much those parameters affect the certified values and uncertainty of the CRMs. In particular, a well-established orthogonal analysis is essential because it allows for cross-validation and enables methodological improvements.

### Comparison of quantification methods

Table 4 shows a comparison of the DNA quantities estimated by different methods used to validate the certified values of the CRMs. The values obtained via digital PCR were 2.3‒9.2% lower than those obtained by counting for the three CRMs. These differences were within the uncertainties of the CRMs (8.3‒11.2%). Thus, we regarded that the values obtained by counting and digital PCR were considered equivalent. However, LC‒MS produced significantly higher values (by 14‒23%), exceeding the range of uncertainties of the CRMs. We reported a similar observation in our previous study in which values obtained via digital PCR and counting were equivalent to each other but approximately 20% lower than those obtained via UV and LC‒MS for single-stranded M13 DNA [22, 24]. LC‒MS can be used to quantify all DNA molecules regardless of sequence or length (Supplemental Fig. 3), which could lead to higher values. The values obtained via different quantification methods are critically affected by the proportions of impurities included in the CRMs. In this context, our first explanation for the observed differences is the possibility that very small fragments (shorter than 100 bp) were included in the CRMs. Such very short DNAs would be quantified only by UV and LC‒MS but not by digital PCR and counting. The other possibility is that LC‒MS overestimates for unidentified reasons. Nonetheless, it is still possible that both counting and digital PCR fail to measure a certain proportion of valid DNA molecules for unknown reasons. Currently, we cannot ascertain the main factors responsible for these differences because of the lack of a well-validated reference measurement system for DNA.

**Table 4:** Comparison of quantification results by orthogonal methods.

	E1			E1-PSG			S		
CRM	Mean (copy/µL)	Expanded uncertainty (95%, *k* = 2)	*N*	Mean (copy/µL)	Expanded uncertainty (95%, *k* = 2)	*N*	Mean (copy/µL)	Expanded uncertainty (95%, *k* = 2)	*N*
Counting	9.08E + 07	11.2%	10	8.55E + 07	11.1%	10	9.13E + 07	8.3%	10
Digital PCR	7.86E + 07	11.5%	10	8.01E + 07	11.2%	10	8.91E + 07	11.5%	10
LC–MS/MS	1.21E + 08	8.1%	3	1.09E + 08	7.5%	3	1.08E + 08	7.9%	3

## Conclusion

We have successfully pioneered a strategy for developing SI-traceable gene-based DNA reference materials. Leveraging plasmid DNA constructs carrying specific genes of interest as common formats for these materials, we utilized single-molecule counting as a certification method to achieve measurements aligned with the new definition of a mole. Our certified values obtained through single-molecule counting were found to be comparable with those obtained through digital PCR but exhibited up to a 20% reduction compared with the values obtained through mass spectrometry. The higher values observed via mass spectrometry were attributed mainly to the difference in measurand definition and partially to the presence of DNA impurities containing irrelevant sequences or shorter lengths. Although our certification method did not directly discriminate such impurities, we precisely analyzed and accounted for them as uncertainties in the reference materials. We anticipate that our CRMs will serve as valuable control materials for a variety of DNA analyses. They will also serve as superior standards for calibrating other methods and developing standard materials at the substage level for DNA quantification. Furthermore, the utilization of gene-based DNA reference materials in plasmid formats offers additional advantages, as common backbone sequences can facilitate both relative and absolute quantification of other DNAs. This advancement not only enhances the accuracy and reliability of DNA quantification but also opens new avenues for future research and innovations in molecular biology and bioanalytical practices.

## Supplementary Information

Below is the link to the electronic supplementary material.

Supplementary file1 (DOCX 1943 KB)

## Data Availability

The datasets generated during and/or analyzed during the current study are not publicly available due to our institutional policy but are available from the corresponding author on reasonable request.
