# Supplementary material for: Development of gene-in-plasmid DNA reference materials certified by single-molecule counting
Source: Anal Bioanal Chem. 2024 Dec 9;417(12):2489–501. doi: 10.1007/s00216-024-05675-1 (PMC12003533; doi:10.1007/s00216-024-05675-1)
Supplement: Supplementary file 1 — Supplementary file1 (DOCX 1943 KB) [file 216_2024_5675_MOESM1_ESM.docx]

**Supplementary information**

**Supplementary figures**


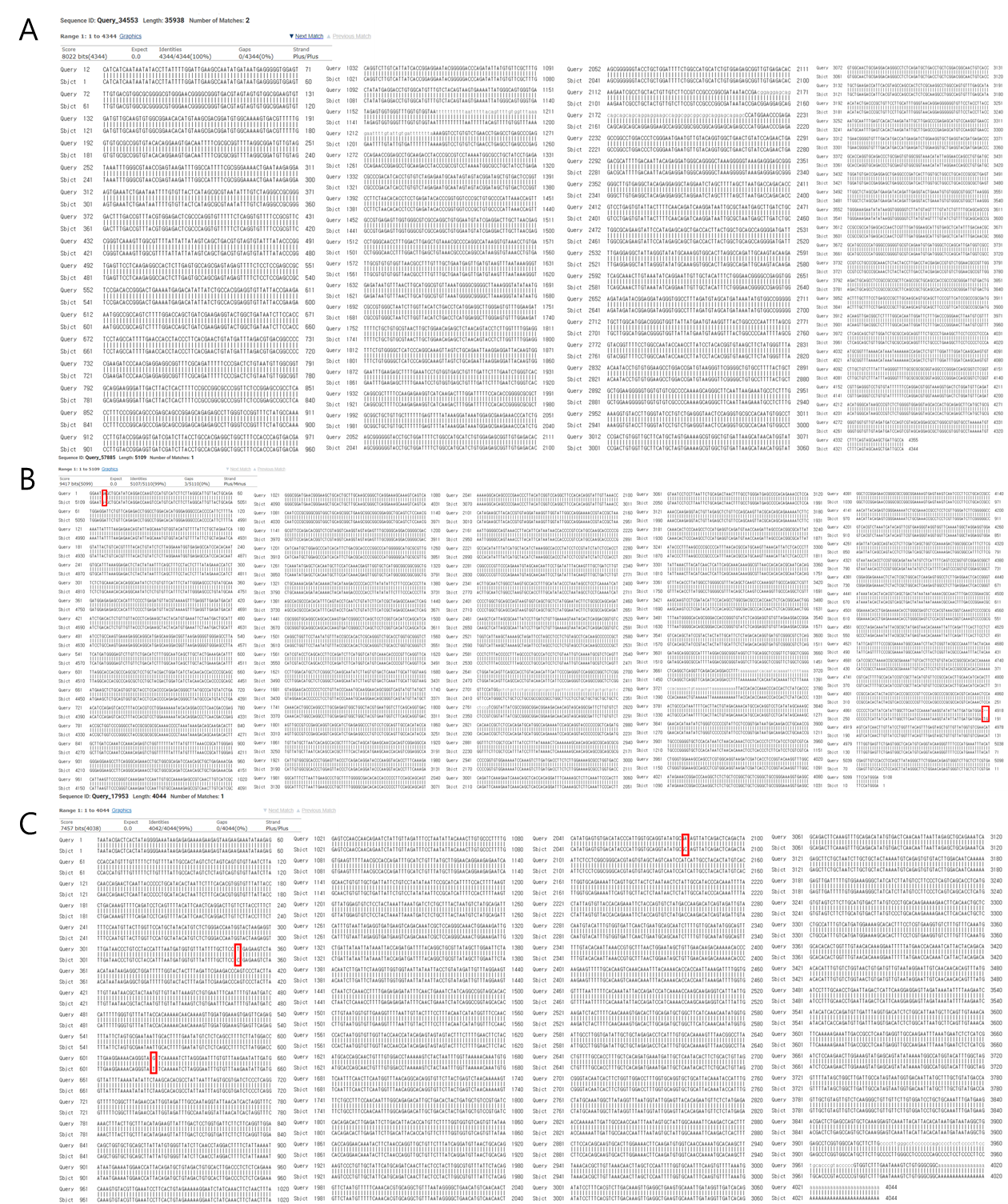


**Supplemental Fig. 1** BLAST results of Sanger sequencing of each insert. a. E1 gene insert, b. E1-PSG, c. S gene insert. Red boxes indicate mismatched base on reference sequences.


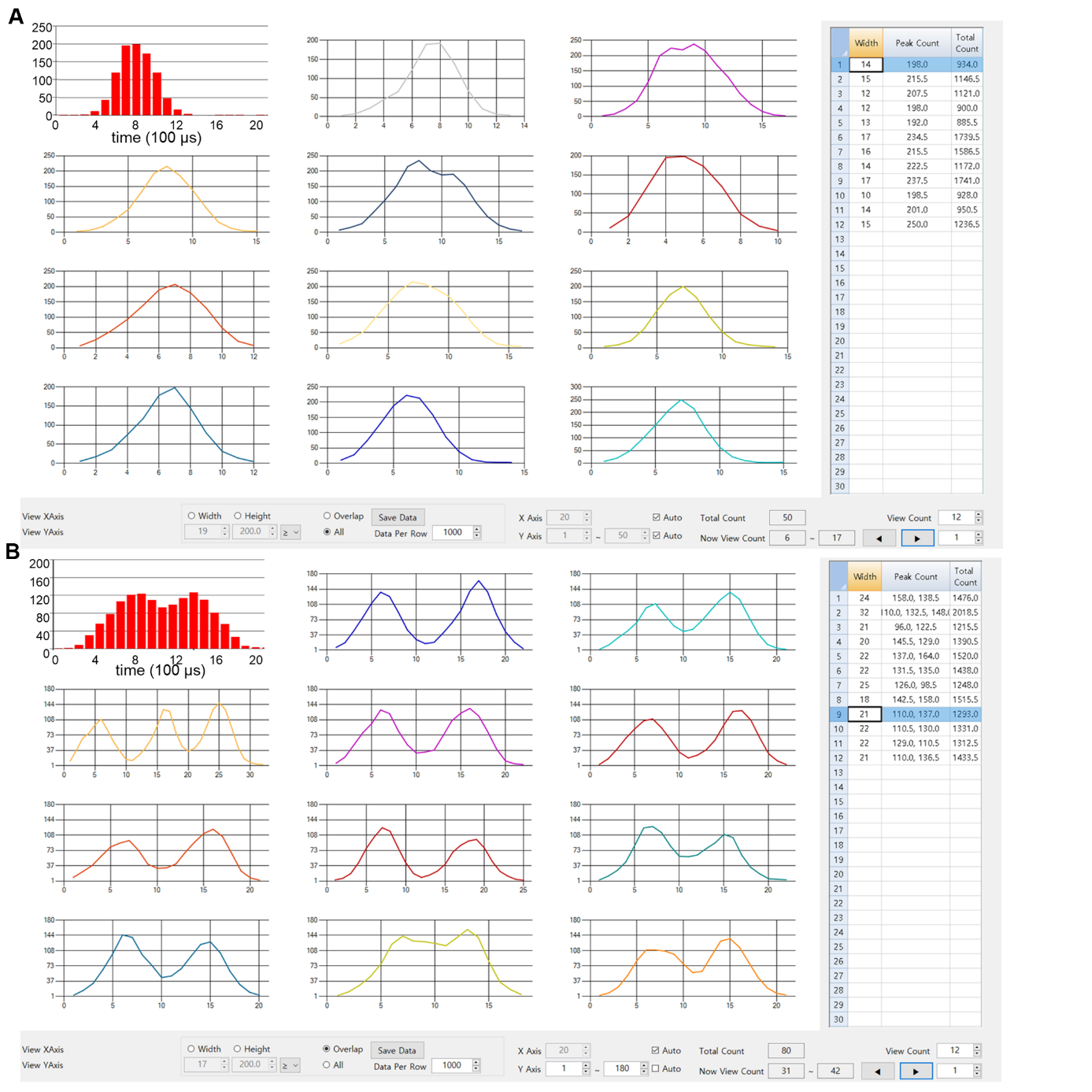


Supplemental Fig. 2 Representative raw data for peaks classified as double molecule counts in certification of the S gene CRM. A. Peaks with higher photon signals than the upper threshold for single molecules. B. Peaks 1.5 times-wider than the average width of single DNA peaks.


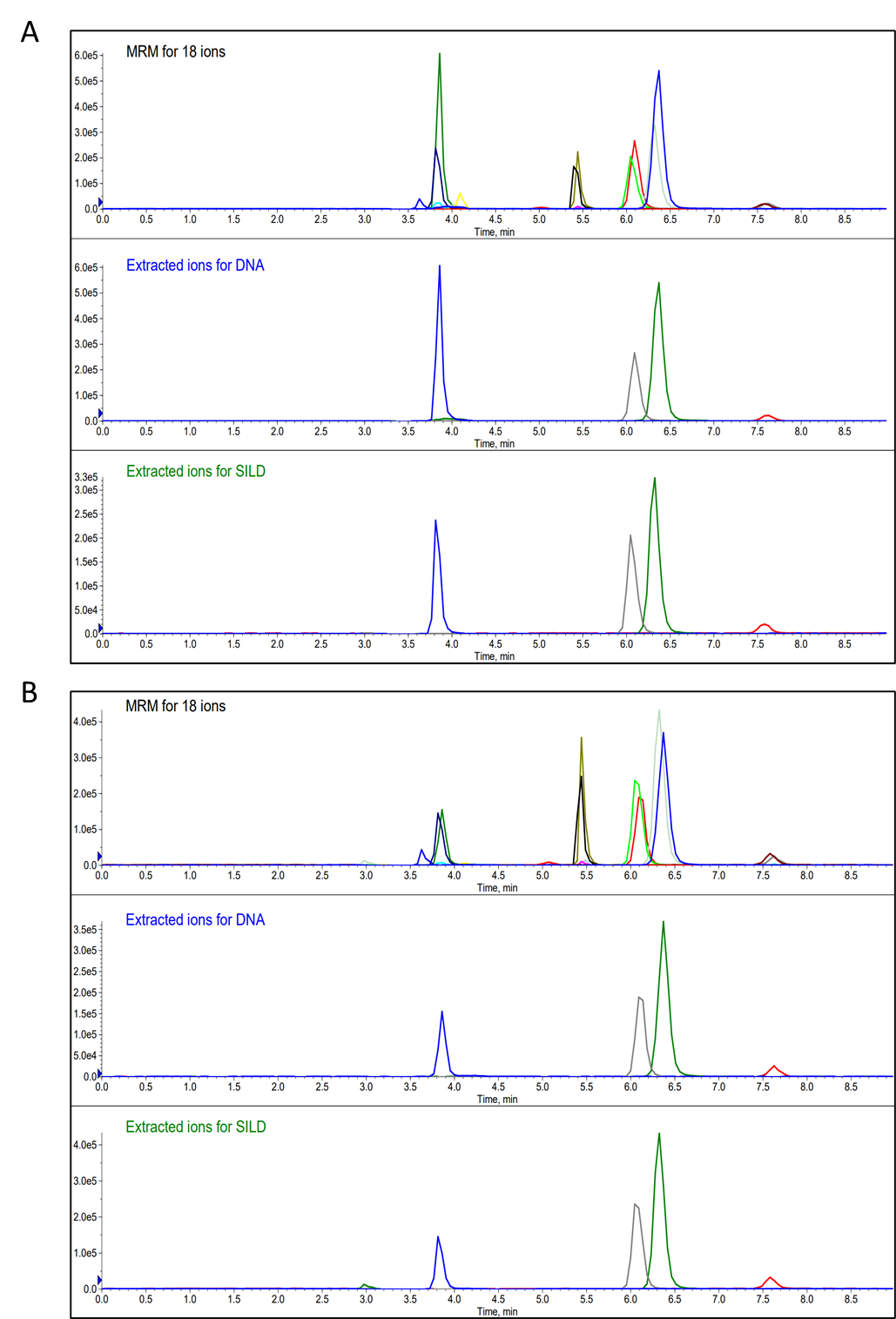


Supplemental Fig. 3 Quantification of the gene-in-plasmid DNA CRMs via ID-MS.

18 nucleosides from DNA and RNA were simultaneously detected in a multiple reaction monitoring (MRM) mode in the ID-MS. Peak areas of extracted ions were normalized by corresponding SILD-ions. dC and SIL-dC: 3.8 min, dG and SIL-dG: 6.1 min, dA and SIL-dA: 6.4 min, dT and SIL-dT: 7.6 min. (A). Mass chromatogram of dNMP standards spiked with SILD internal standards. (B). S gene CRM spiked with SILD internal standards.
